# Investigation of the protective efficacy of *Caesalpinia sappan* and spirulina against ethanol-induced experimental rat gastritis model

**DOI:** 10.1590/acb406725

**Published:** 2025-09-19

**Authors:** İdris Oruç, Zelal Karakoç, Filiz Akduman, Zeynep Oruç, Nazan Baksi, Muzaffer Aydin Ketani

**Affiliations:** 1 Dicle University – Department of Nephrology – Diyarbakir – Turkey.; 2 Dicle University – Department of Clinical Biochemistry – Diyarbakir – Turkey.; 3 Beypazari State Hospital – Department of Pediatrics – Ankara – Turkey.; 4 Dicle University – Department of Oncology – Diyarbakir – Turkey.; 5 Dicle University – Faculty of Veterinary Medicine – Department of Laboratory Animals – Diyarbakir – Turkey.; 6 Dicle University – Department of Histology & Embryology – Diyarbakir – Turkey.

**Keywords:** Spirulina, Gastritis, CD8 Antigens, Major Histocompatibility Complex

## Abstract

**Purpose:**

To evaluate the protective effect of *Caesalpinia sappan* and spirulina against gastritis and determine changes in the expression of CD8, CD68, and major histocompatibility complex (MHC) molecules.

**Methods:**

Gastritis was induced in 24 female Wistar albino rats on the first day using ethanol. The treatment groups were given *C. sappan* (250 mg/kg) and spirulina (400 mg/kg) using oral gavage for five days. Blood and stomach tissue samples of the mice were analyzed.

**Results:**

*Caesalpinia sappan* and spirulina increased CD8 and CD68 expression and tumor necrosis factor-alpha levels thereby decreasing the severity of inflammation. It was found that they simultaneously decreased MHC I and MHC II expressing cells, increased superoxide dismutase levels, whereas malondialdehyde, and myeloperoxidase levels decreased in the treatment group.

**Conclusion:**

This study revealed that *C. sappan* and spirulina can protect gastric mucosa by reducing oxidative stress and inflammation.

## Introduction

Gastritis is characterized by inflammation extending from the gastric mucosa to the serosa. Acute inflammatory changes in the gastric mucosa lead to lesions containing neutrophils or mononuclear cells, resulting in acute erosive gastritis or chronic atrophic gastritis, respectively. Chronic gastritis involves progressive destruction of the gastric mucosa due to persistent inflammation^1^,^2^. The disruption of the gastric mucosal barrier in gastritis triggers neutrophil infiltration and the release of proinflammatory cytokines. During inflammation, reactive oxygen species (ROS) are generated by cytokines such as tumor necrosis factor-α (TNF-α). While ROS regulate cellular functions and enzymatic reactions, excessive ROS production overwhelms the body’s antioxidant defenses–including glutathione (GSH) and superoxide dismutase (SOD)–, leading to oxidative stress. This imbalance contributes to tissue damage, chronic diseases, ulcers, impaired wound healing, and conditions like atherosclerosis, Parkinson’s disease, and malignancies^3^.

The immune system distinguishes self from non-self molecules via major histocompatibility complex (MHC) antigens. MHC class I and II molecules present antigens to CD8+ and CD4+ T cells, respectively, initiating immune responses^4^,^5^. CD68, a macrophage marker, is also expressed by other immune cells (e.g., mast cells, melanocytes), while CD8, a cytotoxic T-cell receptor, is found on natural killer cells, dendritic cells, and monocytes^6^. Macrophages and T lymphocytes play a crucial role in wound healing, yet the distribution of MHC molecules in experimental gastritis models remains underexplored.

Medicinal plants are gaining global interest for their therapeutic potential. *Caesalpinia sappan*, known for its antioxidant and anti-inflammatory properties^7^,^8^, has been traditionally used in Central Asia to treat bleeding gums, anemia, diabetes, and cardiovascular issues, as well as for its antidiarrheal and diuretic effects^9^. It aids vascular repair by modulating endothelial function, inflammation, and redox balance^10^. Similarly, spirulina (*Arthrospira platensis*), a nutrient-rich cyanobacterium, is valued for its sustainability and bioremediation properties^11^. It contains proteins, vitamins, and antioxidants, offering neuroprotective, hepatoprotective, and immunomodulatory benefits^12^–^14^.

This study aimed to evaluate the protective effects of *C. sappan* and spirulina against experimentally induced gastritis in mice, focusing on their anti-inflammatory, antioxidant, and antiapoptotic properties. Additionally, we investigated changes in CD8, CD68, and MHC molecule expression–critical for T-cell-mediated healing–along with alterations in SOD, TNF-α, malondialdehyde (MDA), and myeloperoxidase (MPO) levels.

## Methods

*Caesalpinia sappan* and spirulina (*Arthrospira platensis*) used in this study were procured from Nutrex (Hawaii, United States of America). The taxonomic identities of the species were verified using authoritative databases: spirulina was confirmed via the New York Botanical Garden, while *C. sappan* (WFO ID: wfo-0000214430) was validated through the World Flora Online. Fresh daily doses were prepared and stored at 4°C throughout the study period to ensure stability. The study was conducted in the laboratory of Dicle University.

### Experimental animals

The total of 24 female Wistar albino rats (180–200 g) was housed under controlled conditions, maintaining temperature of 22°C ± 3°C, humidity of 50%–55%, and a 12-hour light/dark cycle. The animals had *ad libitum* access to food and water throughout the acclimatization period. To induce experimental gastritis, rats were fasted for 24 hours (with water provided) and then treated with ethanol. The rats were randomly allocated into six experimental groups.

### Experimental groups

- Group 1 (Control): Healthy rats were given 1-mL saline using oral gavage during the study period;
- Group 2 (*C. sappan*): Healthy rats were given 250 mg/kg *C. sappan* in 1-mL saline using oral gavage during the study period^15^;
- Group 3 (Spirulina): Healthy rats were given 400 mg/kg spirulina in 1-mL saline using oral gavage during the study period^16^;
- Group 4 (ethanol): Healthy rats were given 2-mL ethanol using oral gavage on the first day;
- Group 5 (ethanol + *C. sappan*): Healthy rats were given 2-mL ethanol on the first day and then 250 mg/kg *C. sappan* in 1-mL saline using oral gavage during the study period;
- Group 6 (ethanol + spirulina): Healthy rats were given 2-mL ethanol on the first day and then 400 mg/kg spirulina in 1-mL saline using oral gavage during the study period.

At the end of the study period (six days), animals were anesthetized using xylazine–ketamine (10–90 mg/kg) and sacrificed by drawing blood from the heart.

### Tissue harvesting

Gastric tissue samples were collected from the sacrificed animals and fixed by immersion in a formol–alcohol solution for 18 hours. Following fixation, the samples were processed using standard histological procedures, including paraffin embedding. Serial sections of 5-μm thickness were obtained from the paraffin blocks, and histopathological changes were assessed using Crossman’s Triple staining.

### Immunohistochemical procedure

Tissue sections from paraffin blocks were mounted on aminopropyltriethoxysilane-coated adhesive slides, deparaffinized, rehydrated, and rinsed in distilled water. To quench endogenous peroxidase activity, the sections were treated with 3% H_2_O_2_ in methanol for 20 minutes, followed by washing in 0.01 M phosphate-buffered saline (PBS; 4 × 5 min). Antigen retrieval was performed by heating the slides in 0.01 M citrate buffer (pH = 6) at 95°C, followed by gradual cooling. Nonspecific binding was blocked by incubating the sections with Ultra V Block protein-blocking solution (Thermo Fisher Scientific, TA-125UB) for 15 minutes at room temperature (25°C).

Next, the sections were incubated overnight at 4°C with the following primary antibodies diluted at 1:200: mouse monoclonal anti-MHC I (HLA Ab-1, Clone B-D11; Thermo Fisher), anti-MHC II (HLA-DR Ab-1, Clone LN3; Thermo Fisher), anti-CD8 (UCH-T4; Santa Cruz), and anti-CD68 (KP1; Biocare Medical). After incubation, the slides were washed with 0.01 M PBS (4 × 5 min). For signal detection, the sections were treated with 3,3’-diaminobenzidine (DAB; Thermo Fisher) for 4–10 min. Counterstaining was performed using Gill’s hematoxylin for 1 min, followed by rinsing in tap water until a blue hue developed. Finally, the sections were dehydrated through a graded alcohol series, cleared in xylol, and mounted with Entellan.

**Figure 1:**
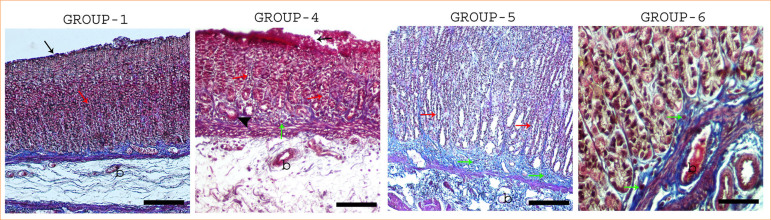
Sections of gastric mucosa of controls and treatment groups (Crossman’s triple stain). Group 1, black arrow: normal mucosa, red arrow: Regular arrangement of tubular fundic glands which are lying perpendicular to the surface with short narrow pits. Group 4, black arrow: impaired mucosal integrity, red arrow: disintegrated glands, arrow head: hemorrhagic area, green arrow: intraepithelial cell infiltration. Group 5, red arrow: disintegrated glands, green arrow: subepithelial cell infiltration. Group 6, green arrow: cells infiltration in stroma. b: blood vessel. Gruop 1 and group 5 figures 25 µm, Group 4 figure 50 µm, Group 6 figure 100 µm

For negative controls, the primary antibodies were replaced with either PBS or normal mouse IgG (Santa Cruz Biotechnology, #sc-2025), followed by the same immunohistochemical protocol (Fig. 1). After staining, samples were examined and imaged using a Nikon Eclipse 400 microscope equipped with a DS-Ri1 digital camera (NIS Elements Imaging Software, v3.10).

b: blood vessel. Source: Elaborated by the authors.

### Immunohistochemical evaluation

The immunohistochemical staining results were semiquantitatively evaluated based on staining intensity, scored as follows: (−) no staining, (+) weak, (++) moderate, and (+++) strong staining^17^. Two independent investigators (MAK and ZK) assessed the immunoreactivity, and the average score of their evaluations was calculated. The localization of MHC I, MHC II, CD8, and CD68 in the stomach was examined using light microscopy at 20×, 40×, and 100× magnifications.

### Biochemical analysis

Prior to sacrifice, blood samples were collected via cardiac puncture under general anesthesia using serum tubes. The samples were transported to the laboratory under a controlled cold chain to maintain stability. Serum levels of TNF-α, MPO, SOD, and MDA were measured in duplicate using commercially available enzyme-linked immunosorbent assay (ELISA) kits. Absorbance readings were obtained using a BS-400 automated spectrophotometer (Mindray, Shenzhen, China).

### Statistical analysis

Statistical Package for the Social Sciences software version 24.0 (IBM, Armonk, NY, United States of America) was used for statistical analysis. Kolmogorov–Smirnov test was used to evaluate the homogeneity of the data. Differences between groups were evaluated using one-way analysis of variance, and Kruskal–Wallis’ test was used as the post hoc test. *P* < 0.05 was considered statistically significant in all analyses.

## Results

### Histopathological findings

**Figure 2:**
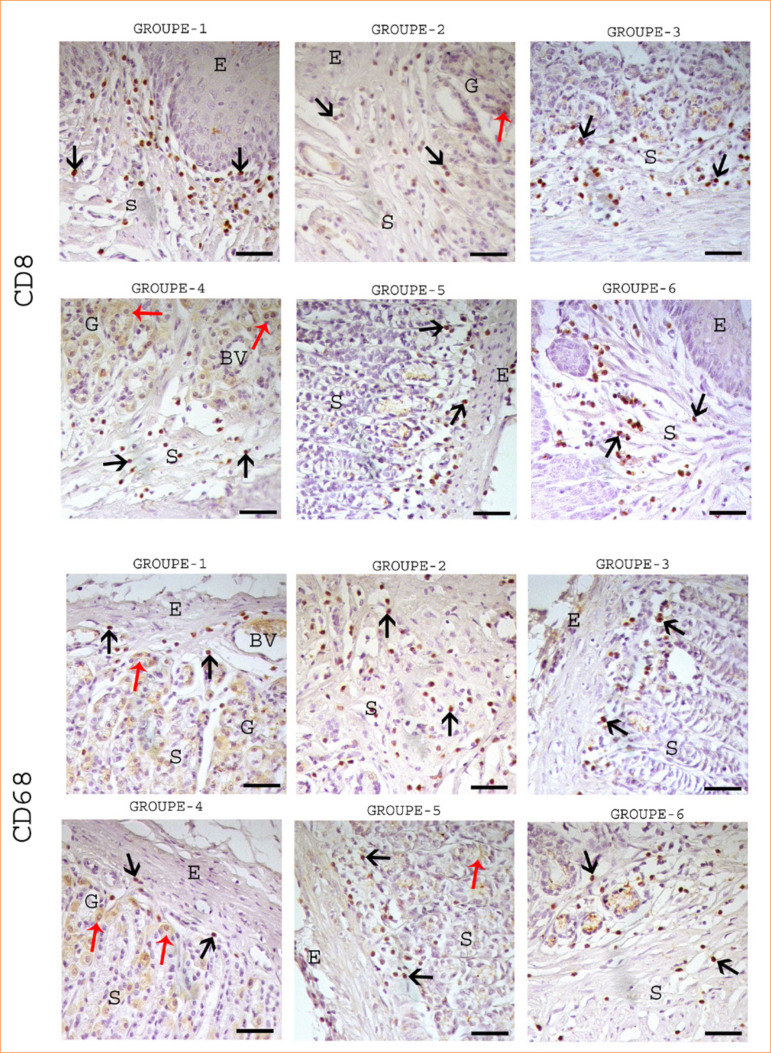
Immunohistochemical staining of CD8 and CD68 in the stomach. Black arrow: positive immunoreactivity in glands cell, red arrow: mitotic activity in cells, E: epithelium, S: stroma, G: glands. All figures 100 µm.

Groups 1, 2, and 3 displayed normal mucosal architecture, characterized by a single layer of prismatic epithelium, a lamina propria densely populated with gastric glands, and a thin lamina muscularis covering the mucosal layer (Fig. 2). In contrast, group 4 exhibited significant epithelial degeneration, with focal disruptions in epithelial integrity. Remnant epithelial cells formed luminal extensions, while the gastric glands appeared dilated and disorganized, accompanied by hemorrhagic areas. Additionally, the lamina propria showed intense inflammatory cell infiltration (Fig. 2).

E: epithelium; S: stroma; G: glands. Source: Elaborated by the authors.

Groups 5 and 6, treated with *C. sappan* and spirulina, respectively, demonstrated reduced mucosal damage, particularly in the apical region. Gastric gland dilatation was markedly diminished, with only mild hemorrhagic foci observed. Notably, these groups exhibited increased mitotic activity in the basal glandular regions of the lamina propria, with group 6 showing the highest proliferative response (Fig. 2).

### Immunohistochemical findings

**Figure 3:**
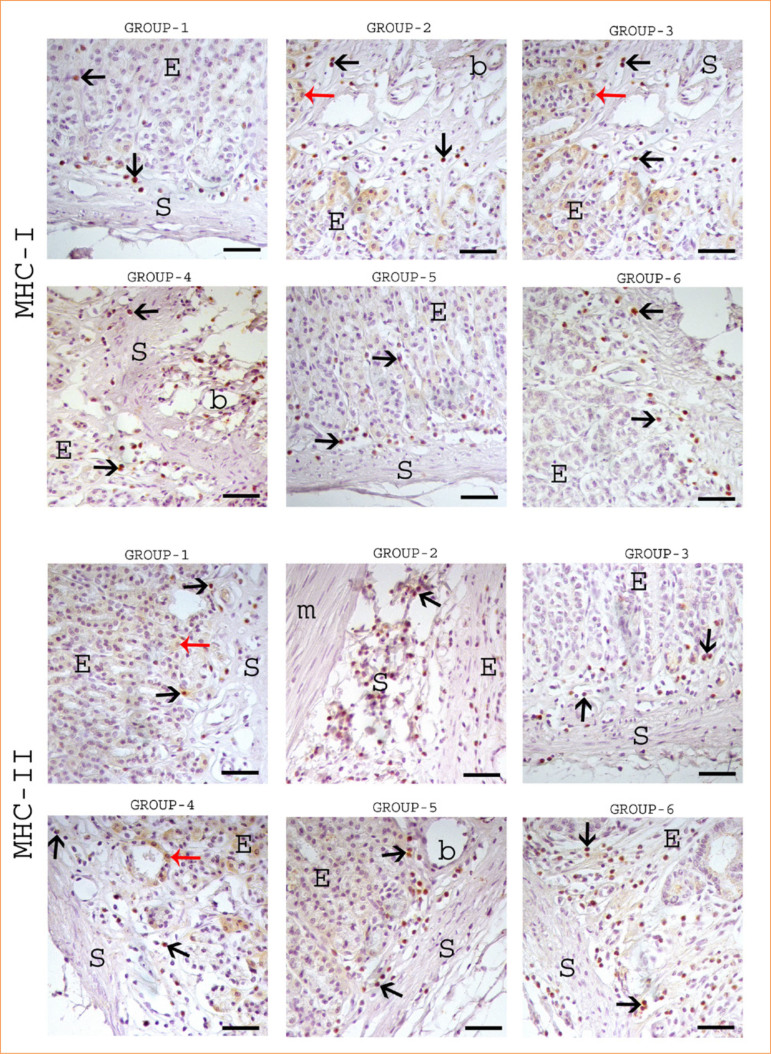
Immunohistochemical staining of MHC-I and MHC-II in the stomach. Black arrow: positive immunoreactivity in glands cell, red arrow: mitotic activity in cells, E: epithelium, S: stroma, G: glands, b: blood vessel, m: musculer layer. All figures 100 µm.

**Figure 4:**
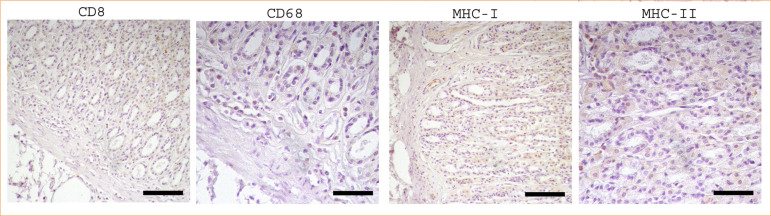
Negative control for CD8, CD68, MHC-I and MHC-II in the stomach in rats. Bar: CD8 and MHC-I figures 50 µm, CD68 and MHC-II figures 100 µm.

Immunohistochemical analysis of CD8, CD68, MHC I, and MHC II in the stomach is summarized in Table 1. Strong CD8 and CD68 immunoreactivity was observed in inflammatory cells within connective tissue regions exhibiting dense cellular infiltrations, with the highest expression seen in group 4 (Fig. 3). MHC I and MHC II-positive intraepithelial lymphocytes were localized in capillary vessel endothelia and connective tissues. Positive immunoreactivity for these markers was widely distributed across the lamina propria in groups 4, 5, and 6 (Fig. 4). However, the number of MHC I- and MHC II-expressing cells was notably lower in groups 5 and 6 compared to group 4 (Table 1).

E: epithelium, S: stroma, G: glands; b: blood vessel; m: muscular layer; MHC: major histocompatibility complex. Source: Elaborated by the authors.

E: epithelium, S: stroma, MHC: major histocompatibility complex. Source: Elaborated by the authors.

**Table 1:** Immunohistochemical expression intensities of CD8, CD68, MHC I and MHC II in stomach.

Groups	CD8	CD68	MHC I	MHC II
Group 1 (control)	++	++	+	+
Group 2 (*Caesalpinia sappan*)	++	++	+	+
Group 3 (spirulina)	++	++	+	+
Group 4 (ethanol)	+++	+++	++	++
Group 5 (ethanol + *Caesalpinia sappan*)	++	++	+	+
Group 6 (ethanol + spirulina)	+	++	++	+

-: no staining; +: weak; ++: moderate; +++: strong; MHC: major histocompatibility complex. Source: Elaborated by the authors.

### Biochemical findings

**Figure 5:**
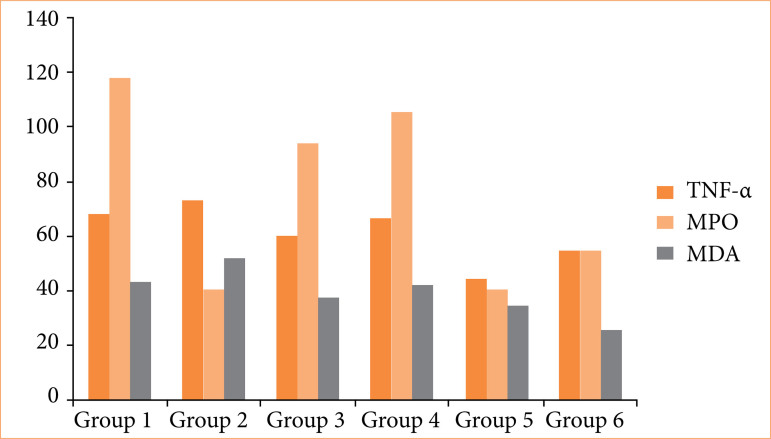
TNF-α (pg mL), MPO (IU/L), and MDA (mmol/L) of ethanol-induced gastritis in rats.

**Figure 6:**
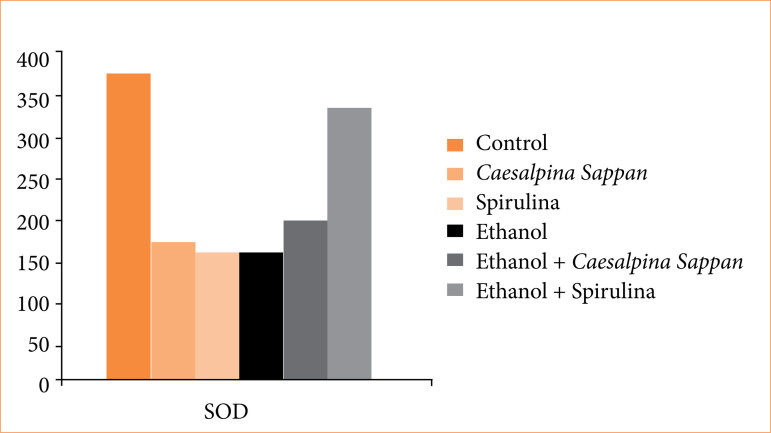
SOD levels (IU/mL) of ethanol-induced gastritis in rats.

Serum TNF-α, MPO, SOD, and MDA levels of the groups are shown in Table 2. Statistically significant difference was not found among the groups in terms of TNF-α, MPO, and MDA levels (Fig. 5). However, a statistically significant difference was found in SOD levels (*p* < 0.05). The highest and the lowest SOD levels were found in groups 1 and 4, respectively (Fig. 6). Intergroup evaluation revealed that SOD level in group 6 was significantly different from groups 2 and 3 (*p* < 0.05).

TNF-α: tumor necrosis factor-α; MPO: myeloperoxidase; MDA: malondialdehyde. Source: Elaborated by the authors.

SOD: superoxide dismutase. Source: Elaborated by the authors.

**Table 2:** Comparison of tumor necrosis factor-α (TNF-α), myeloperoxidase (MPO), superoxide dismutase (SOD) and malondialdehyde (MDA) levels in blood samples from all groupsa.

Groups	TNF-α	MPO	SOD	MDA
Group 1 (control)	65.805 ± 26.33	91.500 ± 105.62	387.950 ± 124.13	46.320 ± 16.31
Group 2 (*Caesalpinia sappan*)	72.450 ± 9.88	29.550 ± 29.48	149.350 ± 60.41	55.820 ± 12.19
Group 3 (spirulina)	64.280 ± 20.88	102.400 ± 68.40	135.700 ± 60.53	33.510 ± 18.11
Group 4 (ethanol)	69.020 ± 16.69	80.300 ± 83.79	143.500 ± 125.77	40.520 ± 17.91
Group 5 (ethanol + *Caesalpinia sappan*)	42.850 ± 14.21	23.150 ± 37.44	188.000 ± 120.59	32.670 ± 22.05
Group 6 (ethanol + spirulina)	48.870 ± 27.94	47.550 ± 33.27	347.000 ± 141.02	16.530 ± 20.79
P değeri	0.138	0.250	0.021	0.237

*ap* are for analysis of variance tests, ranging from *p* = 0.05 to *p* < 0.001. Values are expressed as the mean standard deviation.

## Discussion

The gastric mucosal barrier plays a critical role in protecting the stomach from gastrointestinal damage. While gastric acid, pepsin, and external irritants can compromise mucosal cells, defense mechanisms such as the microcirculatory system, bicarbonate (HCO3-) secretion, prostaglandins, epidermal growth factor synthesis, and epithelial cell remodeling help maintain mucosal integrity. One of the most widely used experimental models to study gastric injury is ethanol-induced acute gastric ulcers^18^. Ethanol triggers gastritis by inducing mucosal hypoxia, vascular damage, and subsequent cellular degeneration and necrosis. Additionally, ethanol contributes to oxidative stress by depleting antioxidant defenses and promoting excessive free radical production^19^.

TNF-α is a potent proinflammatory cytokine that regulates immune responses and inflammation^20^. MDA, a lipid peroxidation product, serves as a biomarker for oxidative stress and tissue damage^3^. MPO indicates neutrophil infiltration in gastric mucosal tissues and is associated with experimental gastric injury^15^. Under physiological conditions, the body maintains oxidative balance through endogenous antioxidants such as GSH and SOD. Among the various markers used to assess oxidative stress and antioxidant activity, SOD is particularly significant due to its role in neutralizing superoxide radicals (*e.g.*, hydroxyl peroxide and molecular oxygen), thereby protecting the gastric mucosa from ROS^3^.

Given the growing interest in plant-derived natural compounds as alternative therapies^21^, studies have explored their potential in mitigating inflammation and oxidative stress. For instance, spirulina platensis supplementation in mice reduced serum TNF-α levels and improved oxidative stress markers–including MDA, nitric oxide, SOD, catalase, reduced GSH, and glutathione peroxidase–in the liver, kidney, and brain^15^. Similarly, *C. sappan* extract administration in rats decreased MPO levels, suggesting its potential as a gastroprotective agent in ulcer treatment.

In the present study, TNF-α, MPO, SOD, and MDA levels were analyzed. While no statistically significant differences were observed in TNF-α, MPO, or MDA levels among the groups, SOD levels showed a significant increase (*p* < 0.05). The highest SOD activity was detected in the control group (group 1) (Fig. 6). Notably, group 6 (ethanol + spirulina) exhibited significantly higher SOD levels compared to group 2 (*C. sappan*) and group 3 (spirulina alone) (Table 2). These findings suggested that both *C. sappan* and spirulina enhance SOD activity, counteracting oxidative stress in experimentally induced gastritis. Although the reductions in TNF-α, MDA, and MPO levels were not statistically significant, the observed trends, combined with the significant rise in SOD, indicate that these natural compounds may exert protective effects on gastric mucosa.

The biochemical, immunohistochemical, and histopathological findings of this study suggested that spirulina exhibits greater efficacy against gastritis than *C. sappan*. The protective effects of spirulina appear to stem from its antioxidant properties, which enhance endogenous enzyme synthesis and reduce lipid peroxidation.

The gastric mucosal barrier, though devoid of resident immune cells, can recruit immune cells via circulation when necessary^5^. Neutrophil infiltration contributes to tissue injury and inflammation by releasing degradative enzymes, particularly in gastric mucosal lesions^22^. Additionally, the lamina propria of the gastric mucosa harbors innate immune components, including CD4+ and CD8+ T cells, plasma cells, macrophages, mast cells, and eosinophils^23^. Macrophages, key players in innate immunity, mediate phagocytosis and cytokine production, with CD68 serving as a specific immunohistochemical marker for these cells^24^. In this study, both *C. sappan* and spirulina reduced inflammation severity and decreased CD8+ and CD68+ cell infiltration in an ethanol-induced gastritis model.

MHC molecules, or human leukocyte antigens, facilitate antigen presentation to T cells. While constitutively expressed, their levels can be upregulated by proinflammatory cytokines (*e.g.*, interferons, TNF) during immune activation^25^,^26^. In our study, ethanol exposure increased MHC I and II immunoreactivity, whereas *C. sappan* and spirulina treatment attenuated this response. This suggested that their antioxidant effects–modulating TNF-α, MDA, MPO, and SOD levels–, along with reduced CD8+ and CD68+ immunoreactivity, may regulate MHC expression during inflammation and healing.

### Study limitations

The small sample size per group, partly due to ethical considerations, may have limited statistical power.

The lack of comparable studies on *C. sappan* and spirulina’s effects on MHC molecules in gastritis models restricts direct literature comparisons for immune marker findings.

## Conclusion

In summary, this study demonstrated that *C. sappan* and spirulina exhibit gastroprotective effects by mitigating oxidative stress and inflammation in acute gastritis. These findings support their traditional use in folk medicine for managing gastrointestinal disorders. Notably, spirulina showed greater efficacy than *C. sappan*, as evidenced by its ability to elevate SOD levels and enhance mitotic activity in glandular epithelial cells. However, further research is necessary to fully elucidate the mechanisms underlying their protective effects against ethanol-induced gastric injury.

## Data Availability

The data will be available upon request.
